# The Use of Chitotriosidase as a Marker of Active Sarcoidosis and in the Diagnosis of Fever of Unknown Origin (FUO)

**DOI:** 10.3390/jcm10225283

**Published:** 2021-11-13

**Authors:** Angela Maria Di Francesco, Elena Verrecchia, Ludovico Luca Sicignano, Maria Grazia Massaro, Daniela Antuzzi, Marcello Covino, Giuliana Pasciuto, Luca Richeldi, Raffaele Manna

**Affiliations:** 1 Institute of Internal Medicine, Periodic Fever and Rare Diseases Research Centre, Policlinico A. Gemelli Foundation IRCCS, 00168 Rome, Italy; difrancesco.angelamaria@gmail.com (A.M.D.F.); elena.verrecchia@policlinicogemelli.it (E.V.); ludovicoluca.sicignano@policlinicogemelli.it (L.L.S.); mg.massaro92@gmail.com (M.G.M.); 2 Institute of Internal Medicine, Catholic University of Sacred Heart, 00168 Rome, Italy; macovino@gmail.com (M.C.); luca.richeldi@unicatt.it (L.R.); 3 Paediatric Clinic, Metabolic Diseases Laboratory, UCSC, Policlinico A. Gemelli Foundation IRCCS, 00168 Rome, Italy; d.antuzzi@gmail.com; 4 Emergency Medicine, Policlinico A. Gemelli Foundation IRCCS, 00168 Rome, Italy; 5 Department of Cardiovascular and Thoracic Sciences, Policlinico A. Gemelli Foundation IRCCS, 00168 Rome, Italy; giuliana.pasciuto@policlinicogemelli.it

**Keywords:** sarcoidosis, chitotriosidase, biomarkers, FUO

## Abstract

Sarcoidosis is a multi-organ inflammatory granulomatosis with a lung-predominant involvement. The aim of this study was to investigate the use of serum chitotriosidase (CHIT1) in patients with fever of unknown origin (FUO); the patients with confirmed diagnosis of active sarcoidosis were compared with ones affected by inactive or treated sarcoidosis. CHIT1 activity was evaluated in 110 patients initially admitted at the hospital as FUOs. The overall performance of CHIT1 for active sarcoidosis diagnosis was assessed by performing an area under the receiver operating characteristic curve analysis (AUROC). The sarcoidosis patients were significantly older than the FUO patients not affected by sarcoidosis (*p* < 0.01). CHIT1 showed a good accuracy as a biomarker for active sarcoidosis in patients explored for FUO (AUROC 0.955; CI 95% 0.895–0.986; *p* < 0.001). A CHIT1 value >90.86 showed 96.8% sensitivity (84.2–99.9) and 85.5% specificity (75–92.8) in discriminating active sarcoidosis from other causes of FUO. CHIT1 significantly discriminated active versus inactive/under treatment sarcoidosis patients (with lower enzyme activity) (ROC analysis, sensitivity: 96.9%, specificity: 94.7%, value >83.01 nmol/mL/h, AUROC: 0.958, 0.862–0.994, *p* < 0.001) compared to ACE (ROC analysis, sensitivity: 25.8%, specificity: 93.7%, value >65 UI/L). In conclusion, CHIT1 is a reliable/sensitive biomarker of active sarcoidosis, with values significantly decreasing in remitted/treated patients. It significantly discriminates active sarcoidosis from FUO patients, providing a useful tool in the diagnosis-assessing process.

## 1. Introduction

Fever of unknown origin (FUO) is defined as a condition of body temperature higher than 38.3 °C on at least two occasions; duration of illness lasting more than 3 weeks or multiple febrile episodes in more than 3 weeks; not immunocompromised; and diagnosis uncertain. Petersdorf and Beeson [1] classified FUOs in four main categories: infectious, malignant/neoplastic, rheumatic/inflammatory, and miscellaneous disorders, essentially the same as included in the Durack and Street review [2]. Sarcoidosis is sometimes found among patients with fever of unknown origin (FUO) [3], which is usually a clinical and diagnostic challenge in internal medicine; it is a multi-systemic inflammatory disease showing granuloma formation in virtually any organ, with a prevalence in the lungs. The diagnosis of sarcoidosis is usually difficult, due to the multi-systemic involvement of the disease requiring a multidisciplinary approach; indeed, there are many different clinical phenotypes (e.g., the Löfgren syndrome is a well-characterized acute form of sarcoidosis) and different prognostic courses, ranging from acute onset or spontaneous patient recovery up to chronic inflammation and fibrosis [4,5].

Traditionally, in FUO patients showing compatible clinical symptoms and non-necrotizing granulomas, the diagnosis of sarcoidosis is established by exclusion of other diseases with similar histological or clinical picture. Chest radiography and CT images are usually indicative of pulmonary disease; however, the diagnosis cannot be exclusively based on imaging and requires a complete clinical and pathological correlation and laboratory tests. 

About potential laboratory tests of diagnostic utility for sarcoidosis, serum levels of angiotensin-converting enzyme (ACE) and soluble IL-2 receptor (sIL-2R), although raised in a substantial proportion of patients with sarcoidosis, showed a low diagnostic value [4,6,7].

In the last decade, serum chitotriosidase (CHIT1 or chitinase-1) levels have attracted growing attention as a potential biomarker for sarcoidosis [8,9,10,11]. Chitinases have a role in both innate and adaptive immunity, since the mammalian enzymes protect the organism against chitin-containing pathogens (e.g., house dust mites, fungi, parasites) [12,13]. There are many lines of evidence reporting that CHIT1 is elevated in sarcoidosis patients compared to healthy controls and that the enzyme levels correlate with the severity of the disease and with the response to corticosteroid therapy [8,9,14].

In the present study, we used CHIT1 as a sensitive biomarker of macrophage activation among patients affected by FUO. Furthermore, we addressed the question whether the value of CHIT1 could discriminate a patient with sarcoidosis in active phase from a patient with sarcoidosis in a remission/inactive state.

## 2. Patients and Methods

### 2.1. Study Population and Study Protocol

The investigation was performed on patients routinely referred to outpatient/day cases or inpatients at the hospital of the A. Gemelli Foundation of Rome (Italy) from January 2013 to January 2020. 

We selected patients admitted to the hospital with fever higher than 38.3 °C lasting more than 3 weeks and who were already studied twice with specific tests, according to Pedersdof and Beeson, modified by Durack and Stret [1,2].

The assessment of sarcoidosis activity was performed every 3 months by image evaluation of lymph nodes, acute phase reactants, and functional pulmonary test as appropriate. All patients with sarcoidosis received a diagnosis histologically confirmed by biopsy, in accordance with the international guidelines [15]. Active sarcoidosis patients showed local or general clinical signs of inflammation and laboratory tests indicative of active inflammation. The diagnosis of inactive sarcoidosis was based on improvement of clinical findings and normalization of acute phase reactants.

In this study, ACE was assayed only after the CHIT1 assay in those patients of the FUO group when suspected to be active sarcoidosis patients.

During this time, 110 patients were recruited. Patients from Group 3 included 11 patients from Group 1 in remission after treatment and 8 patients from the sarcoidosis outpatients follow-up. Informed consent was obtained from all in- and outpatients.

### 2.2. Diagnosis and Measurements

The FUO patients were diagnosed according to specific guidelines and current literature [1,2,16].

### 2.3. Patient Stratification

#### 2.3.1. Active Sarcoidosis Patients

The activity of sarcoidosis was evaluated according to clinical and laboratory exams, i.e., presence of acute phase reactants before steroid treatment. Of the total 110 patients, 33 (28 females and 5 males, age range 30–84 years) were newly diagnosed for active sarcoidosis without any previous treatment (treatment-naive patients) and assigned as Study Group 1 (Table 1). 

#### 2.3.2. FUO Patients

The second group of 69 patients (32 females and 37 males, age in the range 14–82 years) comprised patients with FUO without sarcoidosis (Table 2). For these patients, CHIT1 activity was not monitored during the follow-up but was quantified only at the time of diagnosis, because this parameter was considered sufficient alone to define the biomarker specificity. 

#### 2.3.3. Inactive/Remitted Sarcoidosis Patients

The inactivity of sarcoidosis was evaluated in cases of reduction/normalization of acute phase reactants after steroid treatment. A third group of 19 sarcoidosis patients (of whom 11 belonged to the first group and 8 were diagnosed either in our hospital or in other institutions, age range 25–80 years) was characterized by either inactive or under treatment disease in follow-up in our hospital (Study Group 3, Table 3).

#### 2.3.4. Laboratory Measurements

Plasma CHIT1 and ACE levels were measured from EDTA peripheral blood tests serially collected along the clinical course from all patients involved in the study. Plasma ACE concentration was quantified using a sandwich enzyme-linked immunosorbent assay (ELISA; R&D Systems, Minneapolis, MN, USA). Data were expressed as UI/L. CHIT1 activity was determined by a fluorometric method [17]. CHIT1 activity was expressed in nmol/mL/h of fluorescent 4-methyllumbelliferone delivered by patient plasma enzyme. All the CHIT1 determinations were acquired in the same step to avoid the effect of even small changes in laboratory methodology among different assays. CHIT1 was determined by incubating 5 µL of plasma with 10 µL of 0.022 mmol/L 4-methylumbelliferyl-β-D-N,N′,N″-triacetylchitotrioside (Sigma- Germany) dissolved in citrate/phosphate buffer 0.1/0.2 M at pH 5.2 for 15 min at 37 °C (modified from [17]). After incubation, the reaction was stopped with 1.5 mL of 0.17 M glycine/NaOH buffer at pH 10.6. Fluorescence was measured with a fluorescence spectrometer (LS 45, PerkinElmer®) at excitation and emission wavelengths of 360 nm and 460 nm, respectively. All readings were made against the internal blank with the plasma of the same patient. A standard curve had been previously prepared using the fluorescence of 4-methylumbelliferone.

#### 2.3.5. Statistical Analysis

Continuous, not normally distributed, variables are presented as median [interquartile range] and compared by Mann–Whitney U test. Categorical variables are presented as absolute number (%) and compared by chi-square test (with Yates correction and Fisher test as appropriate). In the case of missing data, a pairwise deletion method was applied.

The overall performance of CHIT1 and ACE in discriminating between active sarcoidosis and other FUO and between sarcoidosis before and after treatment were assessed by performing an area under receiver operating characteristic curve analysis (AUROC). The best discriminating value for each test was assessed by Youden index J. Sensitivity and specificity for diagnosis were assessed by ROC analysis and presented as “value (95% confidence interval)”. AUROCs were compared by the DeLong method. A two-sided *p*-value of 0.05 or less was considered significant. The analysis was made by SPSS v25 (IBM—Armonk, NY, USA).

#### 2.3.6. Sample Size

A good estimation of sensitivity and specificity for a diagnostic test by ROC curve analysis could be obtained with a minimum sample of 100 cases with at least 50% of them being positive. Although our sample is of sufficient overall size, it could be slightly underpowered for a conclusive sensitivity and specificity estimation.

## 3. Results

From the total of 110 patients recruited, 33 had active sarcoidosis (Table 1), 69 had FUO (Table 2), and 19 had inactive sarcoidosis (Table 3). Of the latter ones, 11 patients were already included in Table 1, because they had initially active sarcoidosis and then after treatment were described as inactive sarcoidosis and, thus, included also in Table 3. Further, eight patients from the outpatients sarcoidosis follow-up were added to the third group of inactive sarcoidosis patients. 

The larger part of active sarcoidosis patients showed mediastinal localization, while minorities had skin lesions (6%), eye lesions (6%), and hepatic lesions (9%).

Among the FUO patients, the group with miscellaneous disorders [2] was predominant (55.07%), followed by Fabry disease (15.94%), autoinflammatory disease (11.59%), Still’s disease (5.80%), undifferentiated connective tissue disease (4.35%), fibromyalgia (2.89%), and vasculitis (4.35%).

### 3.1. Active Sarcoidosis versus FUO Patients

Thirty-three patients diagnosed with active sarcoidosis and 69 individuals with FUO were included in the study. Among the group of active sarcoidosis (*n* = 33), 10 were treated and included in a “remission” group with other treated outpatients (*n* = 19). The active sarcoidosis group consisted of 28 females (84.8%) and five males (15.2%). In the FUO group, we had 46.4% females and 53.6% males. Therefore, we observed a significant sex distribution in the two groups, with a prevalence of sarcoidosis diagnosis in females compared to males (*p* < 0.01) as already reported in the literature [18]. Between the two study groups, the mean age of sarcoidosis and FUO patients was 62.9 ± 11.9 and 46.8 ± 18.8 years, respectively, showing a significant age increase (*p* < 0.01) in the first group compared to FUO patients. 

In the active sarcoidosis subset, the CHIT1 levels (202.6 ± 56. 2 nmol/mL/h) were confirmed to be significantly higher than ACE levels (52.8 ± 59.9 UI/L) as already reported [8,10,11]. In the active sarcoidosis group, when compared to FUO patients, CHIT1 values showed a significant accuracy in discriminating active sarcoidosis from other diseases (AUROC: 0.955, CI 95%: 0.895–0.986, *p* < 0.001) (Table 4).

**Figure 1:**
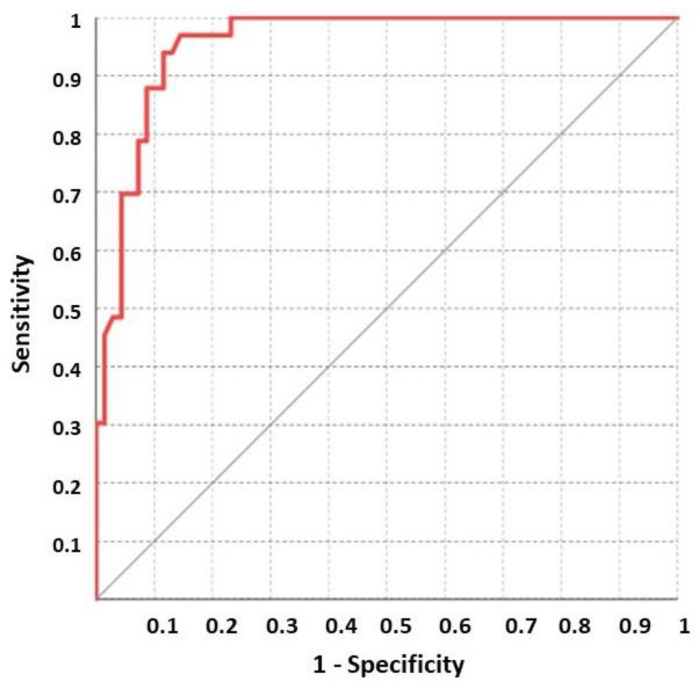
ROC analysis on CHIT1 between active sarcoidosis and FUO patients.

Furthermore, in the ROC analysis, a CHIT1 value >90.86 showed a sensitivity and specificity of 96.8% (84.2–99.9) and 85.5% (75.0–92.8), respectively. These data suggest that CHIT1 discriminates active sarcoidosis patients from other diseases in a specific and sensitive manner (Figure 1).

### 3.2. Active versus Inactive Sarcoidosis Patients

The active sarcoidosis group (before therapy) was compared to patients in remission/under treatment (indicated as “after therapy”) to address whether CHIT1 versus ACE could discriminate between the different phases of the disease. 

No significant difference was observed in the age distribution between the two groups (before therapy and after therapy, median age 63.0 (56.5–74.5) and 65.0 (56.0–71.0) years, respectively; *p* = 0.89).

Furthermore, there was no significant difference in ACE levels between the two groups (before therapy, 34.0 (20.0–66.0) UI/L; after therapy, 37.0 (26.2–62.0) UI/L; *p* = 0.87), whereas CHIT1 was significantly higher before treatment compared to after treatment groups (213.1 (157.8–244.2) and 30.8 (8.4–54.5) nmol/mL/h, respectively; *p* < 0.01).

**Figure 2:**
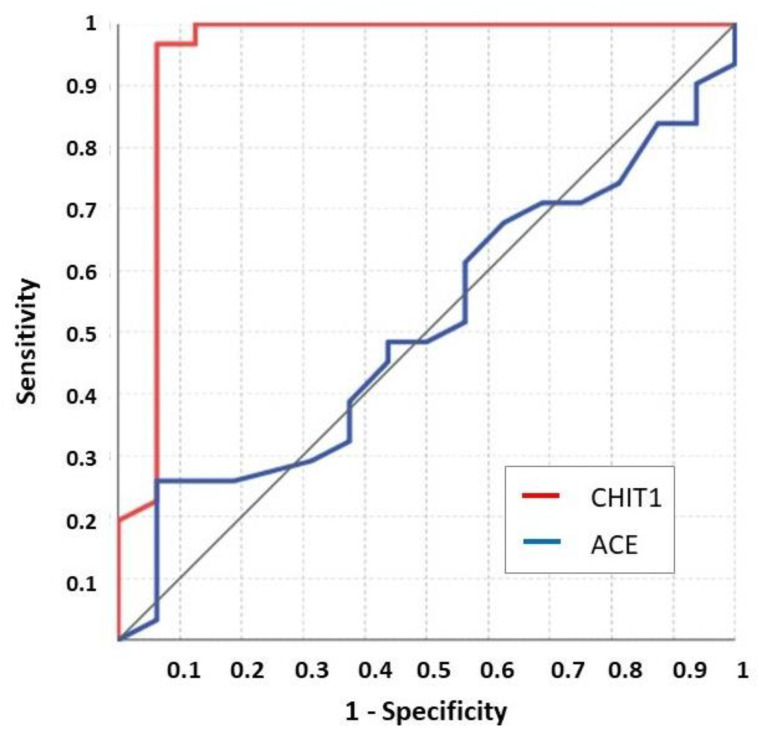
ROC analysis on CHIT1 and ACE values between before and after treatment sarcoidosis patients.

The ROC analysis confirmed that ACE cannot distinguish between sarcoidosis patients before and after therapy (ACE value >65 UI/L showed a sensitivity as low as 25.8% (11.9–44.6) and a specificity of 93.7% (69.8–99.8); AUC = 0.507 (0.357–0.656); *p* = 0.93) (Figure 2).

On the other hand, the ROC analysis confirmed that CHIT1 distinguishes between before and after therapy sarcoidosis patients. In fact, a CHIT1 value >83.01 nmol/mL/h showed a sensitivity and specificity of 96.9% (84.2–99.9) and 94.7% (74.0–99.9), respectively (with AUROC = 0.958 (0.862–0.994); *p* < 0.001) (Figure 2). A special note is warranted for patient no. 14, clinically stable but with modestly high ACE and CHIT1 values, obtained after periodic steroid treatment.

## 4. Discussion

The FUO definition was introduced for research and diagnosis purposes. It includes a wide number of diseases; therefore, the mix of cases may affect the results, but this is the “real life” scenario. The number of FUO patients in this series is comparable to other previously published series, and the male/female ratio distribution is balanced.

Despite the high sensitivity and specificity of CHIT1, its use has never been introduced in routine analysis during sarcoidosis management; however, it could be included as a simple tool for FUO work-up to address granulomatous diseases as we use autoantibodies as tools to address autoimmune diseases.

Table 2 includes a group of patients with FUO which reflects the high clinical variety of this condition; patients within this group showing high CHIT1 values are patients with elevated macrophagic activities as observed in Still’s disease, autoinflammatory disease, large vasculitis, and Gaucher disease.

Indeed, many diseases, including sarcoidosis, show fever at onset. Sarcoidosis is a multi-organ disorder of unknown cause; thus, sarcoidosis patients may present to clinicians of different specialties. The final diagnosis is established through clinical–radiological findings supported by histological evidence of non-caseating epithelioid cell granulomas [19]. Furthermore, due to many variables, such as ethnicity, duration of illness, site and extent of organ involvement, and activity of the granulomatous process, sarcoidosis shows either different modes of clinical presentation or different types of disease onset. As for clinical presentation, there are several types: asymptomatic sarcoidosis (30−50%), without specific constitutional symptoms (in about one-third of patients), or symptomatic sarcoidosis with symptoms related to specific organ involvement. As for the onset of the disease, both acute (showing fever among the symptoms) and chronic (more insidious onset) sarcoidosis are described [19]. In the present study, all sarcoidosis patients were diagnosed according to the international guidelines, confirmed by histology [15]. 

CHIT1 is mainly produced by activated macrophages both in normal and inflammatory conditions [20,21]; therefore, it can be significantly increased in a wide range of diseases involving the macrophages or microglial hyperactivation, such as Gaucher disease, tuberculosis, sarcoidosis, malaria, leishmaniosis, beta thalassemia, multiple sclerosis, and Alzheimer’s disease [20,22,23,24]. CHIT1 is a member of family 18 of the glycosyl hydrolases. This chitinase binds and degrades chitin, one of the most abundant biopolymers in nature and an essential structural component of arthropods [24]. Plasma CHIT1 is considered a biomarker of macrophage activation [25,26], although about 4–6% of the Caucasian population is homozygous for the common null allele responsible for enzyme inactivation [27,28]. CHIT1 is receiving attention as a potential biomarker of sarcoidosis compared to ACE [8,9,10,11]. The potential role of CHIT1 during the diagnostic process of active sarcoidosis patients was further reinforced by recent reports correlating the enzyme’s levels with disease activity, severity, and multi-organ dissemination. Indeed, CHIT1 activity tended to be reduced by steroid therapy [11]. It was recently shown that active sarcoidosis could be differentiated from the inactive one through an algorithm involving the association of plasma CHIT1 activity, ACE levels, and high-sensitivity C-reactive protein (hs-CRP) levels [10]. 

In the present study, a CHIT1 value >90.86 nmol/mL/h differentiated between active sarcoidosis and FUO patients not affected by sarcoidosis with high sensitivity and specificity [96.8% (84.2–99.9) and 85.5% (75.0–92.8), respectively]. We also showed that CHIT1 significantly discriminated between active and under remission sarcoidosis disease as already reported [10], with a sensitivity and specificity of 96.9% (84.2–99.9) and 94.7% (74.099.9), respectively, and AUROC = 0.958 (0.862–0.994) (*p* < 0.001). Elevated values of CHIT1 were observed in those cases with larger localization disease involvement, such as in mediastinal involvement, lymph node involvement, or global systemic involvement.

The observation about patient no. 14, clinically stable with moderate CHIT1 values, suggests a mild activity stabilized by periodic steroid treatment.

In clinical practice, the diagnostic process of FUO is a real challenge for clinicians, since it constitutes a group of over 200 unrelated medical conditions within the differential diagnosis with the common feature of long-lasting fever. Often, FUOs remain an intriguing diagnostic challenge caused by infections (36% of cases), malignancy (19% of cases), inflammatory diseases (19% of cases), or other miscellaneous causes (19%), often difficult to diagnose in spite of extensive medical experience and emerging new technologies; indeed, in a variable percentage of cases, no cause is recognized [29]. Thus, the classical diagnostic methodology for FUO patients is to focus on hallmark clinical features characteristic of each disorder and to proceed through exclusions by searching for both potential diagnostic clues and specific and sensitive laboratory tools to avoid expensive and time-consuming testing.

The peculiar case mix series of the patient cohort from this study may be explained by the selection process operating in our hospital. First of all, this specific internal medicine department is particularly devoted to rare diseases, such as lysosomal and autoinflammatory disorders. Furthermore, admission to the ward is the result of a pre-selection of patients in the ER department after multiple consultations, which sees patients with suspected cancer referred to the department of oncology. 

Therefore, every single FUO case needs to be studied intelligently, and the clinical diagnosis should be guided by the clinical presentation, integrated by image and laboratory tests.

Usually, the detection of ACE levels is also used in the diagnostic and monitoring processes of sarcoidosis patients, but the role of this biomarker is contradictory in real life; however, a correlation between ACE levels and the dynamics of the granuloma burden has been shown [30]. Since the ACE concentrations were higher in non-ACEIs than in the ACEIs group, the appropriateness of ordering serum ACE during renin–angiotensin–aldosterone system inhibitor therapy has been debated [31]. Moreover, when we compared ACE with CHIT1 in our study group, we obtained a low sensitivity (26%) of ACE versus CHIT1, confirming the previous literature [8,9,10,11], and in these case series, the variation range of ACE was narrowed with respect to CHIT1, with the consequent risk of either potentially missing patients with low-activity sarcoidosis or producing false negative results. On the other hand, CHIT1 appeared as an interesting potential biomarker of sarcoidosis activity/severity during the diagnostic process, as high CHIT1 values are indicative of the disease in its active phase, whereas the enzyme activity lowers under treatment or in the remission state of the disease. Furthermore, among other laboratory tests, CHIT1 presents appealing features, being at the same time of simple execution, non-invasive (made on serum or plasma samples), and low-cost, making it feasible to monitor disease activity during steroid tapering and withdrawal.

A limitation of this study concerns the size of the population, since it was a monocentric study in a center devoted to rare diseases and with a specific case mix; however, the message from this study is that the non-invasive and rapid CHIT1 test can represent a specific and sensitive diagnostic tool to discriminate active sarcoidosis patients in the FUO group, contributing in a decisive way to finalizing the diagnostic process.

## 5. Conclusions

When a patient is considered to have a FUO, this represents a real challenge in the internal medicine setting, requiring experience and diagnostic tools to achieve a final diagnosis, which is essential to the specific treatment and, hopefully, improvement for the patient.

Based on the lines of evidence shown in the present study and on the issues previously discussed, we propose that CHIT1 can provide clinicians dealing with pitfalls inherent to FUOs with a powerful and sensitive diagnostic tool. 

We propose to include the CHIT1 test in the initial work-up on FUO patients when macrophage activation is suspected. Therefore, assaying CHIT1 activity during the diagnosis-making process will make an important contribution to the selection of the group of fever-presenting diseases with macrophage activation, such as sarcoidosis, similarly to the use of CHIT1 in other macrophage diseases with different degrees of benignity.

## Figures and Tables

**Table 1:** Study Group 1 (Active sarcoidosis patients).

Patient	Age (Years)	Sex	ACE (n.v. < 64 UI/L)	CHIT1 (n.v.: 4–80 nmol/mL/h)	Site of Biopsy	ExtraP.	Pulmonary Stage
1 *	63	F	309	218.15	Mediastinal adenopathy	No-EP	IV
2	84	F	N.D.	247.36	Mediastinal adenopathy	No-EP	IV
3	63	F	20	147.62	Mediastinal adenopathy	No-EP	I
4 *	63	F	15	109.20	Adenopathic, splenic	Splenic	I
5	54	F	42	234.00	Lymph node/mediastinal adenopathy	No-EP	II
6	58	M	6	218.,56	Liver, supraclavicular adenopathy	No-EP	III
7	75	F	16	254.45	Liver	Liver, bone	II
8	61	F	66	249.44	Submandibular adenopathy	Liver, spleen, and abdominal lymph nodes	I
9	30	M	32	102.13	Liver	Liver and spleen	No-P
10	83	F	N.D.	213.14	Mediastinal adenopathy	Supraclavicular adenopathies	I
11	76	F	51	244.02	Mediastinal adenopathy	No-EP	II
12 *	65	F	20	110.48	Skin	Skin	I
13 *	56	F	34	150.96	Mediastinal adenopathy	No-EP	II
14 *	74	F	62	246.00	Mediastinal adenopathy	Skin	II
15 *	63	F	147	251.95	Skin and mediastinal adenopathy	No-EP	I
16	53	F	29	121.33	Mediastinal adenopathy	No-EP	II
17	59	F	31	210.22	Mediastinal adenopathy	No-EP	II
18 *	76	F	52	335.68	Groin adenopathy	No-EP	I
19	68	M	3	207.29	Periorbital	Orbital	I
20	76	F	30	207.29	Periorbital	Orbital	I
21	64	F	76	251.95	Abdominal adenopathy	No-EP	No-P
22	57	F	26	268.60	Mediastinal adenopathy	Supraclavicular adenopathies	II
23	59	F	99	164.73	Mediastinal adenopathy	No-EP	III
24	48	F	47	224.40	Mediastinal adenopathy	No-EP	I
25 *	45	M	28	145.11	Mediastinal adenopathy	No-EP	I
26	80	F	46	209.38	Mediastinal adenopathy	No-EP	III
27	61	M	20	79.18	Mediastinal adenopathy	Abdominal	No-P
28	50	F	10	224.40	Mediastinal adenopathy	Systemic adenopathies	I
29*	49	F	46	180.59	Laterocervical adenopathy	Systemic adenopathies	I
30	79	F	93	210.20	Axillary adenopathy	Systemic adenopathies	II
31 *	64	F	29	221.07	Mediastinal adenopathy	No-EP	I
32 *	63	F	72	226.07	Laterocervical adenopathy	Systemic adenopathies	I
33	59	F	79	202.29	Mediastinal adenopathy	No-EP	II

* Patients present also in the follow-up group (Study Group 3). N.D.: not determined value. n.v. = normal values of enzymatic activity. The “ExtraP” column contains the extrapulmonary localizations. No-EP indicates absence of extrapulmonary localizations. No-P indicates absence of pulmonary localizations.

**Table 2:** Study Group 2 (FUO patients).

Patient	Sex	Age (Years)	Diagnosis	CHIT1 at Diagnosis (n.v.: 4–80 nmol/mL/h)
34	F	26	Fucosidosis	2.31
35	M	75	Lung cancer (adenocarcinoma)	19.36
36	F	22	Recurrent fevers	25.76
37	F	45	Autoinflammatory disease	1.20
38	F	40	Dystermia	57.06
39	M	60	Recurrent fever with splenomegaly and erythema nodosum	67.08
40	M	67	Aortic stenosis	57.06
41	F	22	PFAPA	82.09
42	F	37	Suspected vasculitis	47.88
43	M	27	Granulomatosis with polyangitis disease	102.13
44	M	17	Still’s disease	33.70
45	M	44	Fabry disease	4.77
46	F	48	Behçet disease	31.60
47	F	63	Undifferentiated connectivitis in family idiopathic lymphedema	192.27
48	F	63	Recurrent peritonitis evolved into mesothelioma	19.08
49	F	37	Periodic fevers	27.01
50	F	45	Crohn’s disease	87.94
51	M	57	Fabry disease	58.73
52	F	50	Autoinflammatory disease	231.50
53	M	33	Suspected hereditary angioedema	4.65
54	F	21	Autoinflammatory disease	65.82
55	F	23	Hypertrophic heart disease	37.86
56	M	62	Hypertrophic heart disease	5.12
57	M	61	Renal insufficiency	4.08
58	M	36	Mitochondriopathy	13.71
59	M	48	Still’s disease with erythema nodosum	90.86
60	M	52	Fabry disease	62.07
61	F	24	Autoinflammatory disease	16.16
62	M	73	Large vessel vasculitis	112.56
63	M	79	BPCO	6.16
64	F	52	Fabry disease	34.94
65	F	37	FMF with erythema nodosum	1.20
66	F	34	Fever in immunodeficiency	47.46
67	F	51	Suspected Gaucher disease	218.15
68	M	59	Variable common immunodeficiency	187.68
69	F	38	Subclinical hyperthyroidism	115.07
70	M	43	Recurring fevers (Castleman disease)	37.03
71	M	38	Autoinflammatory disease	90.45
72	M	48	Lymphoma with sickle cell disease	104.22
73	F	72	Fibromyalgia	24.93
74	M	75	Bile ducts neoplasia	15.33
75	M	74	Fabry disease	8.33
76	M	59	Periodic fever NALP 12	15.16
77	M	72	Fabry disease	3.16
78	M	23	Autoinflammatory disease	10.32
79	F	24	Undifferentiated connectivitis	52.05
80	F	14	Fibromyalgia	10.74
81	M	39	Still’s disease	56.23
82	M	40	Still’s disease	9.84
83	M	57	FMF with lung granulomatous reaction triggered by taking INF alpha	213.97
84	F	70	Suspected Fabry disease	41.62
85	F	32	Fikuchi–Fujmoto disease	3.60
86	M	64	Lymphoproliferating disease with hypogammaglobulinemia	149.29
87	F	62	Hypogammaglobulinemia	89.62
88	F	80	Periodic autoinflammatory fevers	34.94
89	M	82	FUO with hyperthyroidism	30.35
90	M	45	Hypertrophic heart disease	13.60
91	M	54	Fabry disease	5.43
92	F	17	Connectivitis with MEFV mutation	46.63
93	M	27	Fabry disease	4.57
94	F	67	Scleroderma	38.70
95	F	72	COP	1.55
96	M	17	Dystermia	54.14
97	F	30	FMF	0.30
98	F	21	Autoinflammatory disease	82.08
99	M	41	Fabry disease	10.10
100	M	29	Infection in patient under cortisone therapy	7.46
101	M	47	Leucocytoclastic vasculitis of medium-caliber vessels	39.53
102	M	70	Fabry disease	30.35

**Table 3:** Study Group 3 (inactive/after treatment sarcoidosis).

Patient	Age (Years)	Sex	ACE (n.v. < 64 UI/L)	CHIT1 (n.v.: 4–80 nmol/mL/h)	Site of Biopsy	ExtraP.	Pulmonary Stage	Treatment
1 *	63	F	309	60.61	Mediastinal adenopathy	No-EP	IV	P
103	52	F	64	39.53	Skin (erythema nodosum)	Skin	I	Ib, Hy
4 *	64	F	19	43.30	Adenopathic, splenic	Splenic	I	N.T.
12 *	65	F	20	31.19	Skin	Skin	I	P + Chl
13 *	56	F	34	82.01	Mediastinal adenopathy	No-EP	I	P
104	66	F	10	30.77	Mediastinal adenopathy	No-EP	I	N.T.
14 *	74	F	62	244.40	Mediastinal adenopathy	Skin	II	P
15 *	67	F	40	3.53	Skin and mediastinal adenopathy	No-EP	I	MP
105	71	F	N.D.	6.76	Abdominal adenopathy	No-EP	III	P
18 *	76	F	N.D.	23.00	Groin adenopathy	No-EP	I	D
106	66	M	65	43.14	Mediastinal adenopathy	Liver	I	N.T.
107	80	F	26	8.40	Mediastinal adenopathy	No-EP	I	N.T.
108	25	F	52	27.00	Laterocervical adenopathy	Laterocervical adenopathy	I	N.T.
109	67	M	62	27.40	Mediastinal adenopathy	Systemic adenopathies	I	N.T.
25 *	45	M	28	65.40	Mediastinal adenopathy	No-EP	I	P
29 *	49	F	46	<2	Laterocervical adenopathy	Systemic adenopathies	I	F/V
110	74	M	27	30.40	Lung	No-EP	I	N.T.
31 *	64	F	29	54.47	Mediastinal adenopathy	No-EP	I	N.T.
32 *	65	F	N.D.	6.93	Laterocervical adenopathy	Systemic adenopathies	I	N.T.

* Patients present also in the active sarcoidosis group (Study Group 1). N.D.: not determined value. n.v. = normal values of enzymatic activity. The “ExtraP” column contains the extrapulmonary localizations. No-EP indicates absence of extrapulmonary localizations. Treatment column—Chl: chloroquine; D: deflazacort; F/V: fluticasone/vilanterol; Hy: hydrocortisone; Ib; ibuprofen; P: prednisone; MP: methylprednisone; N.T.: no treatment.

**Table 4:** Demographic data and biomarker values in the active sarcoidosis group compared to the other FUO group. Continuous variables are presented as mean ± standard deviation; categorical data are presented as absolute number (%). ^§^ t-test for unpaired data; ^ç^ chi-square test.

	All Patients (*n* = 102)	Active Sarcoidosis (*n* = 33)	Other FUO (*n* = 69)	*p*-Value
Age (years)	58.0 (40–67)	63.0 (56.5–74.5)	45.0 (31.0–62.5)	<0.01 ^§^
Sex (Male)	42 (41.2)	5 (15.2)	37 (53.6)	<0.01 ^ç^
ACE (UI/L)	/	34.0 (26.0–62.0)	/	/
CHIT1 (nmol/mL/h)	54.4 (17.6–172.6)	213.1 (157–244.2)	34.9 (9.9– 66.5)	<0.01 ^§^

## Data Availability

The data presented in this study are shown in Table 1, Table 2 and Table 3.

## Abbreviations

ACE	angiotensin-converting enzyme
ACEIs	angiotensin-converting enzyme inhibitors
AUROC	area under the receiver operating characteristics
CHIT1	chitotriosidase or chitinase-1
FUO	fever of unknown origin
N.D.	not determined.
n.v.	normal value
